# Supplementary material for: Vascular risk is not associated with PET measures of Alzheimer's disease neuropathology among cognitively normal older adults
Source: Neuroimage Rep. 2021 Nov 29;1(4):100068. doi: 10.1016/j.ynirp.2021.100068 (PMC8682073; doi:10.1016/j.ynirp.2021.100068)
Supplement: Multimedia component 1 [file mmc1.docx]

**Supplementary Material**

Principal component analysis (PCA)

We converted each of the eight vascular risk-related measurements (BMI, MAP, HDL, LDL, total cholesterol, triglycerides, fasting glucose, smoking status [current smoker vs. not]) in the larger BLSA data set (n=1588 participants) to *z*-scores. We then split the data into training (n=794 participants) and testing (n=794 participants) sets. We verified that the training and testing sets had comparable distributions for age, sex, and the vascular variables by comparing continuous variables using t-tests and categorical variables using Fisher’s exact test. We did not find any statistically significant differences between training and testing sets. We performed PCA separately on each set to compute principal components in the training set (PC_train_) and in the testing set (PC_test_). We oriented the PCs (and the corresponding scores) such that LDL had a positive loading on each component. We kept the first three components in each data set. The first three components explained 70.3% and 72.1% of the variance in the training and testing sets, respectively. The loadings for each principal component were similar between the training and testing sets (Supplementary Table 1). To quantify the level of similarity, we calculated scores for each participant in the testing set by projecting their measurements onto PC_train_, and assessed the Pearson’s correlation between the scores based on PC_train_ and scores based on PC_test_ for each participant in the testing set. We found high correlations along each principal component (Pearson’s correlation = 0.90 along 1^st^ component, 0.89 along 2^nd^ component, and 0.98 along 3^rd^ component; all p < 0.001) (Supplementary Figure 1). Based on these results, we concluded that PCA of these 8 vascular risk-related measurements yields sufficiently replicable results. The principal components computed in the training set were used to calculate scores for each of the 87 participants included in this study. The difference in the distribution of the scores among these 87 participants and the distribution observed in the training set was not statistically significant for any PC (Kolmogorov-Smirnov test all p > 0.3), suggesting that participants in the BLSA tau PET substudy have vascular risk profiles that are comparable to those observed in the larger BLSA sample (Supplementary Figure 2).

**Supplementary Table 1.** Principal component (PC) loadings in the training and testing sets.

|  | **Training** | | | **Testing** | | |
| --- | --- | --- | --- | --- | --- | --- |
|  | **PC 1** | **PC 2** | **PC 3** | **PC 1** | **PC 2** | **PC 3** |
| BMI | -0.168 | 0.468 | -0.145 | -0.337 | 0.31 | -0.216 |
| MAP | -0.103 | 0.147 | -0.925 | -0.132 | 0.151 | -0.865 |
| HDL | -0.329 | 0.383 | 0.284 | -0.516 | 0.238 | 0.3 |
| LDL | 0.582 | 0.328 | 0.028 | 0.373 | 0.565 | 0.042 |
| Cholesterol | 0.657 | 0.242 | -0.06 | 0.492 | 0.5 | -0.022 |
| Triglycerides | -0.025 | 0.529 | 0.195 | -0.295 | 0.464 | 0.305 |
| Glucose | -0.286 | 0.409 | -0.007 | -0.367 | 0.198 | -0.144 |
| Smoking | -0.004 | 0.005 | 0.003 | -0.005 | 0.001 | 0.001 |
| *Variance explained* | 28.5% | 27.9% | 14% | 29.7% | 27.5% | 15% |


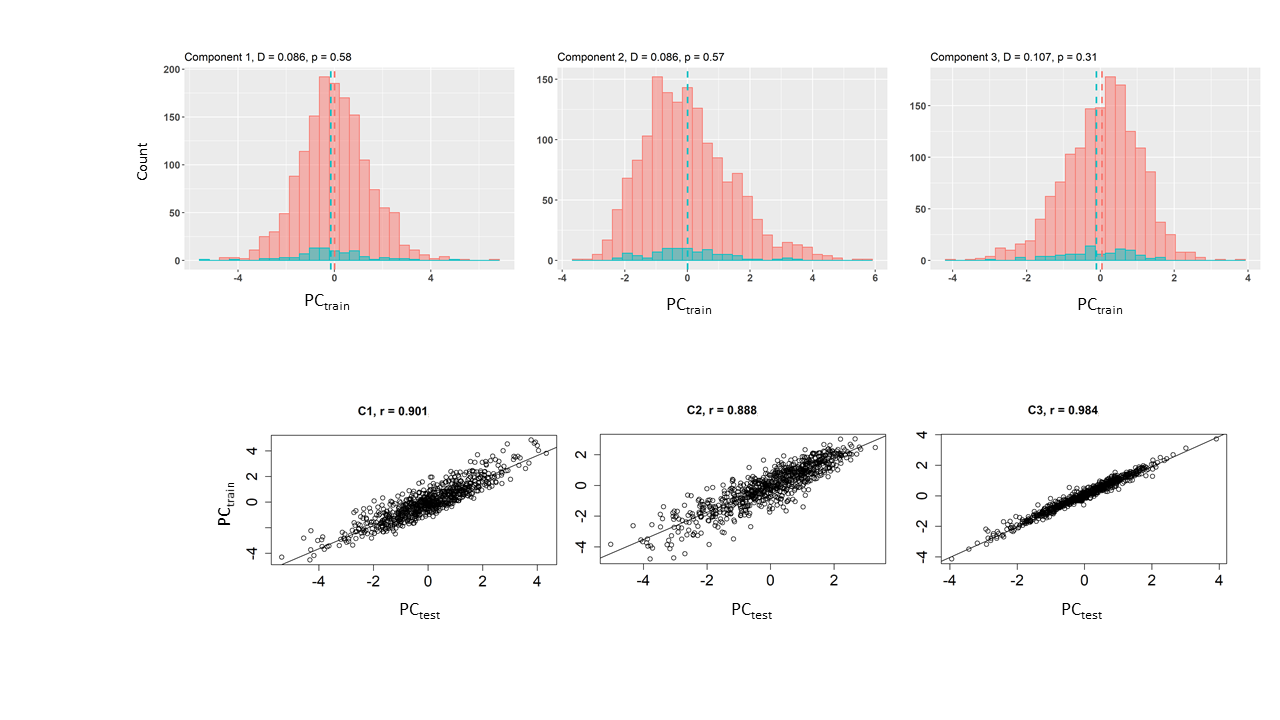


**Supplementary Figure 1.** Comparison of the component scores generated across the two data sets. Correlations are between scores based on the projection of test data onto PC_train_ and scores from PCA_test_.


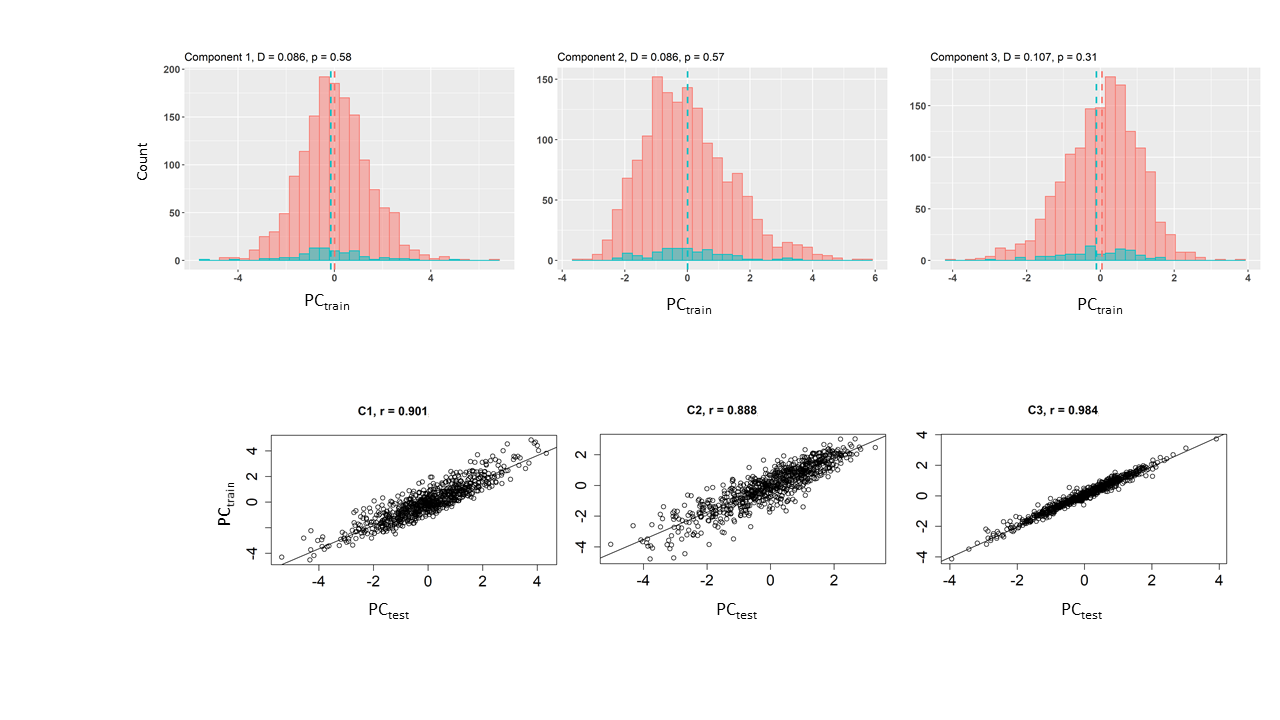


**Supplementary Figure 2.** Histograms showing the scores for the 1501 non-FTP PET BLSA participants (pink) and the 87 FTP PET participants (blue). Scores are based on PC_train_. The vertical dashed lines represent the group means. The Kolmogorov-Smirnov test statistic (D) and its corresponding p value are indicated above each panel.


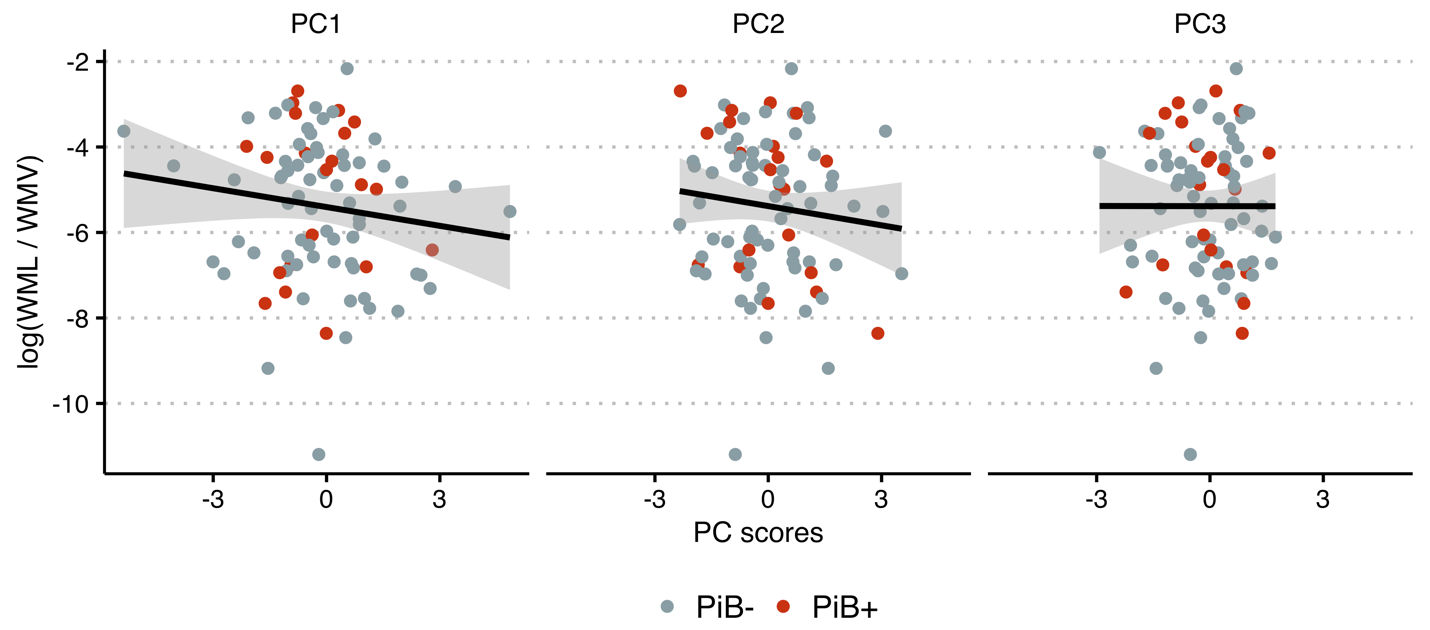


**Supplementary Figure 3.** Log-transformed white matter lesion load (WML) to white matter volume (WMV) ratio versus principal component (PC) scores. Color indicates amyloid-β status determined using ^11^C-Pittsburgh compound B (PiB) positron emission tomography.


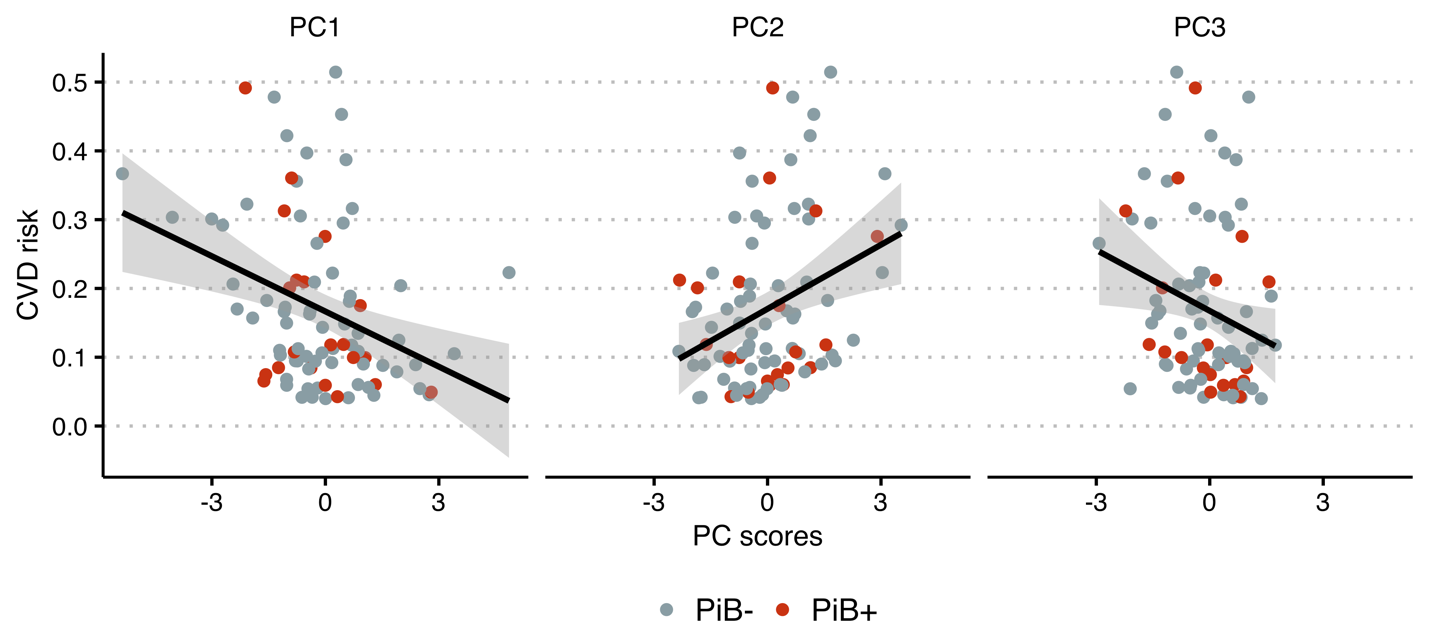


**Supplementary Figure 4.** 10-year cardiovascular disease (CVD) risk versus principal component (PC) scores. Color indicates amyloid-β status determined using ^11^C-Pittsburgh compound B (PiB) positron emission tomography.


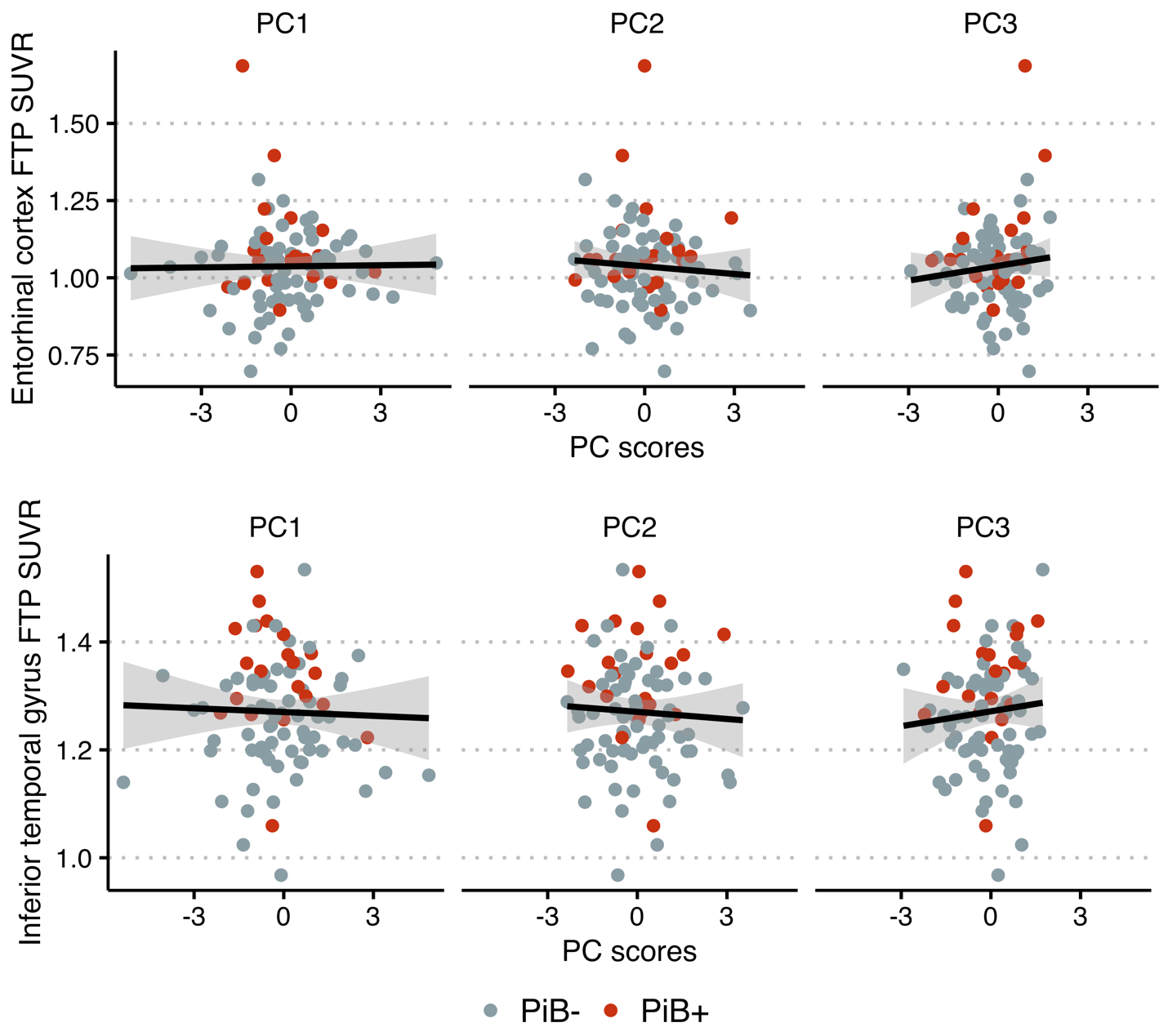


**Supplementary Figure 5.** Scatter plots show no relationship in cognitively normal older adults between principal component (PC) scores and FTP SUVR in the entorhinal cortex (left) and inferior temporal gyrus (right). Color indicates amyloid-β status determined using ^11^C-Pittsburgh compound B (PiB) positron emission tomography.


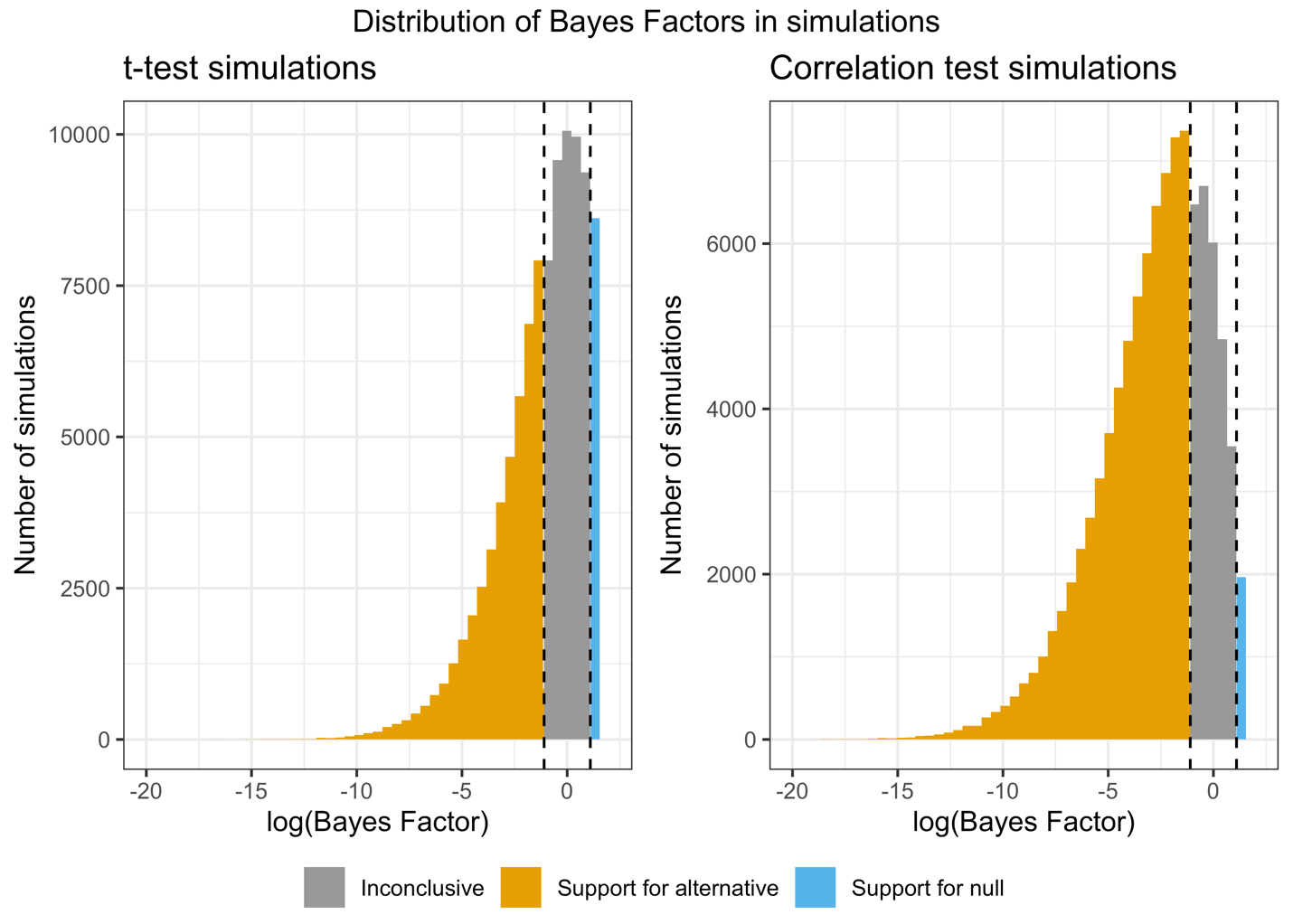


**Supplementary Figure 6.** Distribution of Bayes factors calculated in simulations where data were generated according to the alternative hypothesis. In t-test simulations, the alternative hypothesis was that the effect size (Cohen’s *d*) was 0.5. In correlation test simulations, the alternative hypothesis was that the correlation was 0.3. Since data were generated according to the alternative hypothesis, concluding that the data provide evidence towards the null (i.e., effect size or correlation equals zero) rather than the alternative hypothesis would be erroneous. These simulations demonstrate that the probability of such erroneous conclusions (as indicated in blue) is quite low for both the t-tests and the correlation tests: 9% and 2%, respectively.
